# Supplementary material for: Angle-programmed tendril-like trajectories enable a multifunctional gripper with ultradelicacy, ultrastrength, and ultraprecision
Source: Nat Commun. 2023 Aug 2;14:4625. doi: 10.1038/s41467-023-39741-6 (PMC10397260; doi:10.1038/s41467-023-39741-6)
Supplement: Supplementary file 1 — Supplementary Information [file 41467_2023_39741_MOESM1_ESM.pdf]

Supplementary Information for

Angle-programmed tendril-like trajectories enable a multifunctional gripper with ultradelicacy, ultrastrength, and ultraprecision

Yaoye Hong[1], Yao Zhao[1], Joseph Berman[2], Yinding Chi[1], Yanbin Li[1], He Huang[3,4], Jie Yin[1,*]

[1] Department of Mechanical and Aerospace Engineering, North Carolina State University, Raleigh, NC 27695, USA.

[2] Department of Electrical and Computer Engineering, North Carolina State University, Raleigh, NC 27695, USA.

[3] UNC-NC State Joint Department of Biomedical Engineering, North Carolina State University, Raleigh, NC, USA.

[4] UNC-NC State Joint Department of Biomedical Engineering, University of North Carolina at Chapel Hill, Chapel Hill, NC, USA.

[*]To whom correspondence may be addressed. Email: jyin8@ncsu.edu

Supplementary Note 1. Comparison with existing grippers

Supplementary Note 2. Control of the deploying gripper

Supplementary Note 3. Program of the grasping trajectory

Supplementary Note 4. Grasping mechanism

Supplementary Table 1-Table 2

Supplementary Fig. 1 - Fig. 10

Supplementary References

Supplementary Note 1. Comparison with existing grippers

Comparison between our work and other grippers. The advantages of this work compared to previously reported soft grippers is summarized in the Supplementary Table 1 and discussed below.

Comparison with noninvasive grippers. We start by comparing our gripper with several representative soft and noninvasive grippers. For example, the kirigami gripper based on a rhombic shell[1] (Yang et al., Science Robotics 2021, Ref. 9 in the main text) can pick a raspberry using pinching mode. The nondestructive gripper based on an origami polyhedron[2] (Teoh et al., Science Robotics 2018, Ref. 7 in the main text) and hydraulic ribbons[3] (Sinatra et al., Science Robotics 2019, Ref. 3 in the main text) can grasp a jellyfish using enclosing mode. Existing noninvasive grippers can be applied to fragile objects and gelatinous organisms. First, from the perspective of noninvasive feature, none of existing grippers can be applied to any of the high-requirement scenarios in biology (i.e., nondestructively grasping marine life softer than jellyfish) and biomedicine (i.e., noninvasively manipulating a liquid drop), as shown in Supplementary Table 1. Distinctly, our gripper surpasses them in the noninvasive feature. It can nondestructively manipulate a liquid drop with near-zero stiffness, as shown in Fig. 1. This feature arises from programming the grasping trajectory toward the nastic curve in plants. The gradually curling-up trajectory enables an ultra-gentle touch. From the perspective of the strong feature, the payload-to-weight ratio in our work is 16000, two orders magnitude larger than that of other works shown by Supplementary Table 1. This payload-to-weight ratio is of great importance for noninvasive tasks in unstructured environments[4]. The payload-to-weight ratio in Yang et al.'s work[1] is about 222 due to the pinching grasping mode. The ratio in Teoh et al.'s work[2] is less than 100 due to the rotating polyhedron made of rigid thermoplastic. Third, from the perspective of the design simplicity, in Teoh et al.'s work[2], the supporting stents are necessary to rotate the origami polyhedron. Both Teoh et al.'s[2] and Sinatra et al.'s work[3] require the support of bulky external hydraulic systems. In Yang et al.'s work[1], extra design modifications (e.g., extended appendages) are required for grasping a grain of sand or medical pills. In our work, one design can specialize across different tasks. This feature facilitates assisting prostheses in delicate tasks shown in Fig. 1 and Fig. 5, where frequently changing the end effector for different tasks is necessary in existing prostheses.

Comparison with strong grippers. Compared with the reported strongest gripper using a gold layer coupled with shape memory polymers[5] (Roh et al., Science Robotics, 2021, Ref. 17 in the main text) with a payload-to-weight ratio of 6,400, our gripper has a ratio 2.5 times higher (16,000). Compared to all existing strong grippers based on stimuli-responsive materials, suction, fluid-driven rigidity percolation, jamming, adhesive materials and electro-adhesion shown by Fig. 3f and Supplementary Table 1, our gripper has the largest payload-to-weight ratio.

Comparison with universal grippers. Existing universal grippers rely on the suction[6] (Song et al., Advanced Materials 2021, Ref. 5 in the main text), responsive shape memory polymers[7] (Linghu et al., Science Advances 2020, Ref. 16 in the main text), and jamming[8] (Brown et al., PNAS 2010, Ref. 1 in the main text). However, none of them can achieve universal grasping while retaining the noninvasive feature. Specifically, suction cannot preserve the gelatinous organisms during grasping. Shape memory polymers require a high temperature (e.g., 80°C in Linghu et al.'s work[7]) that could harm living organisms, let alone the sacrificed response time. The jamming structure cannot grasp gelatinous organisms, let alone liquid objects.

Comparison with our previous work. From the analytical perspective, in the proof-of-concept gripper demonstration of the previous work[9] (Hong et al., Nature Communications 2022, Ref. 10 in the main text), we experimentally and qualitatively explored the deformation of the gripper. This gripper deformation is not explored through analytical analysis or numerical simulation. It is due to the complex gripper structure, i.e., the coupled polygons composed of curved ribbons. Though the deformation of a single polygon composed of ribbons was explored, the coupled polygons makes the problem challenging. Further, based on the Gauss-Bonnet theorem, the integral of the changeable curvature along the boundary makes it difficult to program and control the grasping trajectory (refer to Supplementary Note 2).

In this work, the new angle-based design allows for simple programming of the grasping trajectory through an angle γ_0 and explicit control of the trajectory through the applied strain ε. To our knowledge, this work first programs and controls the grasping trajectories of soft grippers explicitly. The experimental results are consistent with analytical and numerical analysis.

Programming and controlling the trajectory leads to significantly improved grasping capability, as discussed later.

From the application perspective, our previous work[9] demonstrates a proof-of-concept kirigami gripper based on the curvature-based design, which can nondestructively grasp an egg yolk and a live fish. However, the unprogrammed trajectory inhibits the gripper's potential to specialize across different extreme scenarios in biology and biomedicine.

Herein, we programmably drive artificial trajectories towards nastic curve in nature leads to significantly improved grasping capability. Additionally, the controllable trajectory results in unprecedented performances when integrated with robotic arms and prostheses, which is never explored in our previous work[9].

First, for noninvasive tasks, the tendril-like trajectory enables the new gripper to manipulate a water drop with near-zero stiffness, whereas the previous one stopped at gelatinous organisms. Second, for heavy objects, the curled-up trajectory enables it to handle 16,000 times the weight of itself, which is 16 times higher than the previous one[9]. Additionally, the bending-to-stretching energy evolution yielded by the curling-up trajectory enables a Janus-faced feature, ultrastrong while retaining the ultragentle feature. This bending-to-stretching energy evolution is first harnessed in soft grippers to generate a record-high payload-to-weight ratio. Third, for ultra-thin targets, the gradually curling trajectory enables it to grasp a 2-μm-diameter fiber, 40 times smaller than the previous version (80-μm-diameter)[9]. Fourth, when the gripper is integrated with the robotic arms and the prosthesis that rely on a cost-effective displacement control, the new angle-based design eases the displacement control of the tendril-like trajectory. This makes feedback control unnecessary in delicate tasks. For example, in noninvasively picking a grape from the vine, the success rate is nearly two times higher than the previous gripper[9]. The integration with prostheses and robotic arms is never demonstrated in our previous work[9].

Overall, our new design surpasses the previous one[9] in specializing across different extreme scenarios, including handling extremely soft, ultra-heavy, and ultra-thin/tiny objects. A comparison of the presented work and our previous work[9] is provided in Supplementary Table 2.

Supplementary Note 2. Control of the deploying gripper

Comparison between the curvature-based and angle-based control. The curvature-based design is capable of generating various 3D shapes[9]. However, the inexplicit and nonlinear relationship between the displacement and the Gaussian curvature makes it not suitable for the explicit displacement control. For the morphology induced by the boundary curvature, we arrive at a scaling of the curvature as a function of the in-plane tension: $k_b \sim \sqrt{\tau}$, where k_b and τ denote the curvature of the central point in the boundary ribbon and the corresponding in-plane tension, respectively. For the curvature-dominated boundary[9], the differential equation for the shear tension is given by

$$\frac{d}{ds}\left(\frac{1}{k_b}\left(p_n + \frac{dq}{ds}\right)\right) + k_b q = 0 \tag{1}$$

where s denotes the arclength. p and q are the load due and the transverse shear tension, respectively.

Substituting q using the linear constitutive equation of the bending moment, we find

$$\frac{d}{ds}\left(\frac{1}{k_b}\left(\frac{1}{2}k_b^3 + \frac{d^2(k_b - k_{bo})}{ds^2} + \frac{p_n}{B}\right)\right) - k_b\frac{dk_{bo}}{ds} = 0 \tag{2}$$

The in-plane tension is expressed as

$$\tau = C_1 B(k_b^2 + C_2 + f(k_b, k_{bo})) \tag{3}$$

where τ and k_b denote the in-plane tension and the boundary curvature, respectively. B is the bending rigidity. C_1 and C_2 are constants. $f(k_b, k_{bo})$ is a function related to the boundary curvature k_{bo} in the reference configuration and is zero when the boundary of the precursor is circular.

To resolve this obstacle, based on the Gauss-Bonnet theorem from differential geometry[10], we harness the angle γ_0 instead of the curvature to simplify the displacement control of the morphology during uniaxial stretching. The variation in the boundary angle is simply controlled by the displacement and the relationship is expressed as $\varepsilon = \frac{\sin\left(\frac{\gamma}{2}\right)}{\sin\left(\frac{\gamma_0}{2}\right)} - 1$, as discussed later, where ε and γ are the applied strain and the angle in the boundary, respectively. Thus, the curvature-based

design[9] is suitable for force control and the angle-based design is more explicit for displacement control.

The displacement-controlled morphing of the angle-based design. To better understand the angle-based design of the kirigami gripper in Fig. 1, we first explore how the prescribed intersecting angle γ_0 guides the displacement-controlled morphology of shell-like grasping petals. Supplementary Fig. 1 shows the kirigami design of a fan-shaped 2D petal patterned with parallel cuts enclosed by a V-shaped straight ribbon and a circular boundary ribbon with $\gamma_0 = 120^o$.

Upon uni-axial stretching of the 2D petal, the discrete ribbons pop up to form a shallow shell-like shape with a positive Gaussian curvature K, arising from the increase in the deformed intersecting angle γ (Supplementary Fig. 2b, i). As γ further increases with the applied strain ε, the shell grows with increasing K (Supplementary Fig. 2b, ii). When $\gamma = \gamma_{max} = 180^o$, it arrives at a maximally stretched state, corresponding to a fully deployed shape (Supplementary Fig. 2b, iii). Supplementary Fig. 2c shows that the morphology and curvature of the fully deployed shells can be programmed by γ_0, where a relatively smaller γ_0 generates a larger K due to its larger angle variation $\Delta\gamma = \gamma - \gamma_0$ ($\gamma_0 \leq \gamma \leq 180^o$). Especially, when $\gamma_0 = 180^o$, K remains zero due to $\Delta\gamma = 0$ and the sheet remains flat (Supplementary Fig. 2c, iv).

For different γ_0, the angle-based design presents a simple and explicit relationship between the applied strain ε and γ, i.e.,

$$\varepsilon = \frac{\sin\left(\frac{\gamma}{2}\right)}{\sin\left(\frac{\gamma_0}{2}\right)} - 1 \quad (4)$$

This largely simplifies the complex and implicit control equation in the boundary curvature-based design[9]. Note that when the fan-like precursor is combined with the X-shaped ribbons, this relationship need to be modified using the constant c_r related to the length of the stretching ribbon. Supplementary Fig. 2d shows that γ first increases linearly with ε, followed by a steep rise near the fully deployed state for all the prescribed angles γ_0. The correlation between ΔK and $\Delta\gamma$ can be analytically obtained by modeling each discrete ribbon as Euler elastica[10], which gives

$$\Delta\overline{K} \sim \frac{m_s F(\frac{\pi}{2}, m_s)}{\cos(\frac{\Delta\gamma}{2})} \tag{5}$$

where $\Delta\overline{K} = \Delta K/K_{max}$ with K_{max} being the Gaussian curvature of the fully deployed state, m_s is the elliptic modulus characterizing the buckling shape of discrete ribbons, and F denotes the incomplete elliptic integral of the first kind. The plot of $\Delta\overline{K}$ *vs*. $\Delta\gamma$ in Supplementary Fig. 2e shows that $\Delta\overline{K}$ first increases monotonically with $\Delta\gamma$ and then approaches a plateau when $\Delta\gamma$ approaches its maximum, which is consistent with the experiments. Thus, with Eqs. (4)-(5), γ bridges the gap between K and ε (or displacement) via an explicit relationship. This simplifies the displacement control of the shape-shifting in the gripper and eases the deploying control when it is integrated with prostheses and robotic arms relying on a cost-effective displacement control.

Optimal angle γ_0 from the perspective of morphing. As shown in Supplementary Fig. 2a, when γ_0 =15°, the deformation and the variation in the Gaussian curvature are dominated by the curvature variation in the circular boundary instead of the angle variation in the triangular boundary. The triangular boundary cannot be fully stretched where the circular boundary has been straightened. Thus, the normalized Gaussian curvature in the ultimately stretched shell-like shape has the maximum value near γ_0 = 80°, as shown in Supplementary Fig. 2b. When γ_0 >80°, the length of the triangular boundary is smaller than that of the circular boundary, thereby making γ_0 = 80° a critical point.

Supplementary Note 3. Program of the grasping trajectory

Shape of the discrete ribbons. The shape of the discrete ribbons in the conical and spherical regions can be approximated as Euler Elastica[11] and is expressed as

$$y = \frac{2}{\lambda} E(AM(\lambda s, m), m) - s \quad (6)$$

$$z = \frac{2m}{\lambda} CN(\lambda s, m) \quad (7)$$

where *y* and *z* are the coordinate functions. The discrete ribbons use the arc length coordinate, and s denotes the arc length of the discrete ribbons, where the origins are set at the central point. *E*, *AM*, and *CN* are elliptic functions. *m* represents the elliptic modulus, which characterizes the buckling of the ribbon, as shown in Supplementary Fig. 3b.

Equilibrium in grippers. The force *F* acted on the stretching ribbon of the gripper can be decomposed into F_x and F_y, as shown in Supplementary Fig. 3a. We assume that the bending energy stored in the conical and spherical regions is from the work done by F_y and F_x, respectively. Thus, the work *W* can be expressed as $W = \int Fl \sin\theta \cos\theta \, d\theta$, where θ denotes the angle between *F* and F_y, and the work done by F_x and F_y is equal to each other. When γ_0 is not zero, the bending energy stored in the conical and spherical regions is assumed to be equal to each other. The energy stored in the two representative ribbons of the two regions is used to represent that stored in the corresponding regions. Then, the relationship between the elliptic modulus of the ribbons in conical and spheroidal regions is derived, as shown in Supplementary Fig. 3c. With the deployment of the gripper, m_s increases nearly linearly with m_c. The elliptic modulus m_s describing the buckling shape of discrete ribbons is derived using

$$\frac{2E(\frac{\pi}{2}, m_s)}{F(\frac{\pi}{2}, m_s)} - 1 = \frac{(1+c_b)\sin\left(\frac{\gamma_0}{2}\right) - c_b}{\sin\left(\frac{\gamma_0}{2}\right) + \cos\left(\frac{\gamma_0}{2}\right)} \quad (8)$$

where *E* denotes the incomplete elliptic integral of the second kind, with c_b being a constant related to the buckling of the boundary ribbon. We concentrate on the precursors with $\gamma_0 > 80°$. In 2D precursors with $\gamma_0 < 80°$, the sharp angle can hardly be fully stretched and is constrained by the length of the circular boundary (Supplementary Fig. 2a).

Program and control of the grasping trajectory. We note that all the deploying trajectories of the end effectors are consistent with the Euler spiral[11]. The trajectory curvature linear increases along the arclength coordinate s_t of the trajectory curve. We also note that the arclength s_t increases linearly with the applied strain. Thus, s_t bridges the gap between the applied strain and the trajectory and makes the trajectory controllable. Further, with γ_o increasing, the curvature at the maximum applied strain decreases. Based on the Gauss-Bonnet theorem[10], the variation in the summation of exterior angles is proportional to the integral of the Gaussian curvature. The two principal curvatures are assumed to be equal, i.e., the square root of the Gaussian curvature. Then the varying principal curvature yields the variation in the trajectory curvature. Thus, we arrive at a scaling of the maximum curvature as a function of the variation in the original intersecting angle. Supplementary Fig. 4d-f shows the simulation results of grippers with γ_o =80°, 130°, and 180° are consistent with the experiments, where the maximum tensile strain is small. Moreover, the gripper with γ_o =180° exhibits a U-shape trajectory similar to that of grippers with γ_o =130° and 150°, which may cause wrinkling or failure in grasping ultra-thin objects.

Enclosing volume of petals. For the gripper with γ_o = 80°, the deploying hemispherical petals with an over 2.4 times larger enclosing volume V than that of the shallow-shell petals of the gripper with γ_o = 150° (Supplementary Fig. 4a). We note that for the gripper with γ_o = 80°, V is over 35 times the volume V_o of its undeployed flat state (Supplementary Fig. 4a). Meanwhile, it creates a large accommodation room over 40 times its undeployed volume. The elastic and reversible deployment in the kirigami gripper with minimal occupying volume is in sharp contrast to most undeployable soft grippers, which will facilitate its easy integration with other robotic and prosthesis robotic systems. Consequently, considering the trajectory, the shape of petals, and the volume, γ_o = 80° is optimal for designing the gripper.

Supplementary Note 4. Grasping mechanism

Implications for precision tasks. The unique features in the gripper with γ_0 = 80°, such as close-to-180° grasping angle and curling-up trajectory, account for its precision grasping of ultrathin sheet objects in Fig. 5b-5c. Supplementary Fig. 5a schematically illustrates the thin sheet grasping mechanism. First, when the end effectors touch the substrate surface (Supplementary Fig. 5a, i), the gradual curling in the trajectory facilitates the tip entering the gap between the target and the substrate to perform encapsulating (Supplementary Fig. 5a, ii). Second, α~180° enables the spherical petals to approach the sheet in parallel and insert the end tip in parallel to the sheet into the gap between the sheet and the lying surface. This parallel approaching and encapsulating mode minimizes the horizontal interaction between the petals and the target thin sheet. Note that when the petals are in contact with the surface, α further increases to be closer to 180° before the closure of the petals. Then, the curled-up trajectory (Fig. 2d) ensures the petals lift the target (Supplementary Fig. 5a, iii). It is noteworthy that by virtue of the ω-shaped trajectory, accurate vision control of the traveling routine or distance is not necessary, where the only requirement is to ensure that the end tip of the petal touches the substrate surface. Thus, the parallel encapsulating mode avoids pinching or compressing the target, which facilitates noninvasively grasping a super-thin sheet. Compared to the grippers with larger γ_0, e.g., $\gamma_0 = 130°$ and 150°, their U-shaped trajectories and smaller angle α can hardly grasp the target sheet without compressing the target horizontally. The pinching mode results in the buckling of the thin sheet or a failure of grasping.

Effect of the boundary shape. From the perspective of qualitative design, the upper and boundary curves are set to be curved with a C^2 continuity. The C^2 continuity represents the curve, the first derivative, and the second derivative are continuous (i.e., continuous in curvature). It enables the petals to be near spherical in the deployed state, which facilitates the encapsulating of the target. If the continuity of the upper and lower boundary shape is decreased, it leads a larger gap between ribbons of petals. For example, Supplementary Fig. 4 shows a rhombic precursor with a C^0 continuity. The C^0 continuity represents a continuous curve but with an abrupt slope change. Stretching it generates a gap at the discontinuous point due to the abruptly changing slope. From the perspective of quantitative design, we analytically analyze the effect of the variation in the angle γ and boundary curvature on the Gaussian curvature of the petals during deploying. The

Gauss-Bonnet theorem for each petal is simplified as $\int_{\Omega} K dA = C + \pi - \gamma - \oint_{\partial\Omega}(k_b \sin\varphi) ds$, where K denotes the Gaussian curvature. γ and k_b denote the angle in the petal and the curvature along the boundary of the surface formed by the petal, respectively, as shown by the figure below. φ is the angle between the plane containing the boundary and the normal plane to the discrete ribbon. C is a constant related to the Euler characteristic. Overall, the angle and curvature affect the Gaussian curvature through the term γ and $\oint_{\partial\Omega}(k_b \sin\varphi) ds$, respectively.

As shown by Supplementary Fig. 7, during deployment, the prescribed curvature k_{bo} in the 2D precursor changes to k_b in the deployed gripper, with γ_o changing to γ. Then, we tune the prescribed curvature in 2D precursors, where k_{boT} denotes the tuned prescribed curvature in 2D precursors (Supplementary Fig. 7a). The tuning range is kept within the 30% of the k_{bo}, i.e., $\frac{|k_{boT} - k_{bo}|}{k_{bo}} < 30\%$. For these tuned precursors, the effect of the variation in the curvature term k_b can be neglected compared to that of the variation in γ, (i.e., $\Delta[\oint_{\partial\Omega}(k_b \sin\varphi) ds]/\Delta\gamma < 4\%$). As a result, the limited changes in the prescribed curvature of the upper and lower boundary shape of the precursor barely changes the morphology, if the continuity of the boundary keeps at least C^2.

Effect of the width of the cut. Supplementary Fig. 8 shows the isometric view of the deployed grippers with different thickness/width w_c of cut at the maximum applied strain. To characterize the effect of thickness/width w_c of cut, we define a dimensionless variable w_c/w. w denotes the width of the gripper. In the grippers demonstrated in the main text and Supplementary Fig. 8a, the variable is minimized ($w_c/w = 0.003$). It maximizes the contact area between the petals and the target object to reduce the contact pressure. With w_c/w increasing to 0.025, the empty space becomes larger, as show in Supplementary Fig. 8c-d. First, for noninvasive tasks (e.g., grasping a liquid drop), the increased contact pressure leads to failure of noninvasive grasping the liquid drop. The petals intrude into the drop even with a hydrophobic coating. Second, for delicate tasks, the performance is barely affected by the width w_c of cut, if $w_c << w$. Third, for high-load tasks, the energy increase is predominantly stretching. The maximum tensile stress σ is proportional to the variable w_c/w (i.e., $\sigma \propto w_c/w$). The increased w_c/w decreases the maximum payload of the gripper.

Effect of the aspect ratio of the precursor. Supplementary Fig. 9a shows the dimension of the 2D precursor. We tune the varying length l_v to change its aspect ratio, which further changes the length l with the width w unchanged. This is to retain the gripper's morphological features. Supplementary Fig. 9b-d shows the side view of the deployed grippers with aspect ratio r_a = 1.64, 2.75, and 4, respectively. First, for the grippers with a small aspect ratio r_a, as shown in Supplementary Fig. 9b, the deployed gripper can only form an unclosed shape composed of shallow-shell petals at the maximum applied strain. It results from the decreased ribbon length, which requires a larger bending energy to bend the ribbon in the spherical petals. The increased energy requirement prohibits the formation and the closure of the fully-bending petals. As a result, the gripper fails to grasp gelatinous or tiny objects. Second, with an increased aspect ratio r_a (e.g., r_a = 2.75 in Supplementary Fig. 9c), the decreased bending-energy requirement facilitates the formation of closed spherical petals. We observe that when the ratio r_a vibrate around 2,75, the grasping capability keeps stable. Third, when the aspect ratio increases larger than 4 (e.g., r_a = 4 in Supplementary Fig. 9d), the larger ribbon length makes the two petals meet earlier (i.e., at a smaller applied strain). The petals pinch the target object. This compression makes the gripper unsuitable for noninvasive tasks. Additionally, it prohibits the formation of straightened geodesic network, which further decreases the payload-to-weight ratio.

Regarding delicate tasks. The demonstration for delicate objects (e.g., waterdrop and jellyfish) serve as compelling showcases of the extraordinary delicacy exhibited by our gripper. Furthermore, it highlights the immense potential of our gripper in tackling challenging scenarios encountered in the fields of minimally invasive biology conservation and biomedicine. Achieving ultra-delicate grasping poses a significant challenge due to the uncertainties arising from the dynamic task environment (e.g., wetting conditions, viscosity, and rate of operation) and how the living objects will react to touch (e.g., when grasping gelatinous organisms such as a jellyfish). However, by programming the trajectory of our gripper to emulate the nastic curve found in nature, we have significantly enhanced its capability to handle the uncertainties of unknown and unstructured environments, all without the need for feedback control. Regarding wetting conditions, we pick the waterdrop from a flat surface with commercially available hydrophobic coating (Rust-oleum multi-purpose spray kit). This limitation arises due to the inherent challenges associated with picking up a spreading water droplet from a hydrophilic surface without causing damage or

compromising the integrity of the droplet. Regarding viscosity, our gripper can be applied to fluids and gelatinous organisms with different viscosity, including Newtonian fluids (e.g., a waterdrop in Fig. 1b and Supplementary Movie 1), non-Newtonian fluids (e.g., ketchup in Supplementary Fig. 5f and Supplementary Movie 4), a living jellyfish (Fig. 4b and Supplementary Movie 4). Regarding rate operation, the petals, actuated by the Robotiq 2F-85 gripper, approach the waterdrop at velocities of 65mm/s and 35mm/s (Supplementary Movie 1). Remarkably, we have found that the success rate remains consistently high (76%), even when the velocity is varied within this range. This indicates the robustness and reliability of our gripper's performance across different operating speeds.

Effect of the size of the target. For noninvasive tasks, the gripper is designed to be larger than the object. It enables the gripper to encapsulate the target to minimize the compression exerted on the target. This encapsulating grasping mode is different from various prevalent soft grippers using pinching mode. The pinching mode requires the gripper to compress the target and then use the friction to lift it. The compression makes noninvasively grasping a water drop or gelatinous organisms impossible.

For the tasks without a high requirement on non-invasion, we use a dimensionless parameter l_{tar}/l_g to analyze the variation in performance of the gripper. As shown in Supplementary Fig. 10a, l_{tar} and l_g denote the length of target and length of the undeployed gripper, respectively. With l_{tar}/l_g <0.45, the gripper performance barely changes with the curling trajectory scooping up the target. It enables the formation of a straightened geodesic network. As a result, the stretching energy is utilized to hold the target. With $l_{tar}/l_g >0.45$, the failure in forming a straightened geodesic network leads to a sharp drop in the pulling-out force. Additionally, the gripper has to compress the target and use the friction force to lift the target (Supplementary Fig. 10b). The energy increase in the gripper is predominant bending.

Supplementary Tables

	[1]	[3]	[7]	[14]	[15]	[16]	[17]	[9]	[10]	[5]	[44]	[46]	This work
Grasping mechanism	Jamming	Fluid-driven actuator	Fluid-driven actuator	responsive material	responsive material	responsive material	responsive material	Stretching-induced deformation	Stretching-induced deformation	Suction	Electro-adhesion	Adhesive material	Stretching-induced deformation
Programmable deformation	×	×	✓	×	✓	×	×	×	✓	×	×	×	✓
Programmable and controllable trajectory	×	×	×	×	×	×	×	×	×	×	×	×	✓
Noninvasively manipulate gelatinous organisms	×	✓	✓	×	×	×	×	×	✓	×	×	×	✓
Noninvasively manipulate liquid objects	×	×	×	×	×	×	×	×	×	×	×	×	✓
Gentleness (contact pressure)	Unexplored	0.0455 kPa	Unexplored	Unexplored	Unexplored	Unexplored	Unexplored	Unexplored	Unexplored	-15.7 kPa	Unexplored	2.5 kPa	0.0468 kPa
Strength (maximum payload-to-weight ratio)	Unexplored	Unexplored	<100	380	925	330	6,400	222	1,000	490	1,067	Unexplored	16,000
Delicacy (minimum resolution for ultra-thin objects)	Unexplored	Unexplored	Unexplored	Unexplored	Unexplored	10 μm	3.2 mm	1.5 mm	80 μm	2.5 mm	Unexplored	Unexplored	2 μm
Manipulate gelatinous organisms when integrated onto robotic arms and prostheses	×	✓	✓	×	×	×	×	×	✓	×	×	×	✓
Manipulate liquid objects when integrated onto robotic arms and prostheses	×	×	×	×	×	×	×	×	×	×	×	×	✓

Supplementary Table 1. A comparison between other soft grippers and this work. The top row represents the reference number in the main text.

	Previous work	This work
Programmable deformation	✓	✓
Programmable and controllable trajectory	×	✓
Noninvasively manipulate gelatinous organisms	✓	✓
Noninvasively manipulate liquid objects	×	✓
Gentleness (contact pressure)	Unexplored	0.0468 kPa
Strength (maximum payload-to-weight ratio)	1,000	16,000
Delicacy (minimum resolution for ultra-thin objects)	80 μm	2 μm
Manipulate gelatinous organisms when integrated onto robotic arms and prostheses	×	✓
Manipulate liquid objects when integrated onto robotic arms and prostheses	×	✓

Supplementary Table 2. A comparison between our previous work[9] (Ref. 10 in the main text) and this work

Supplementary Figures

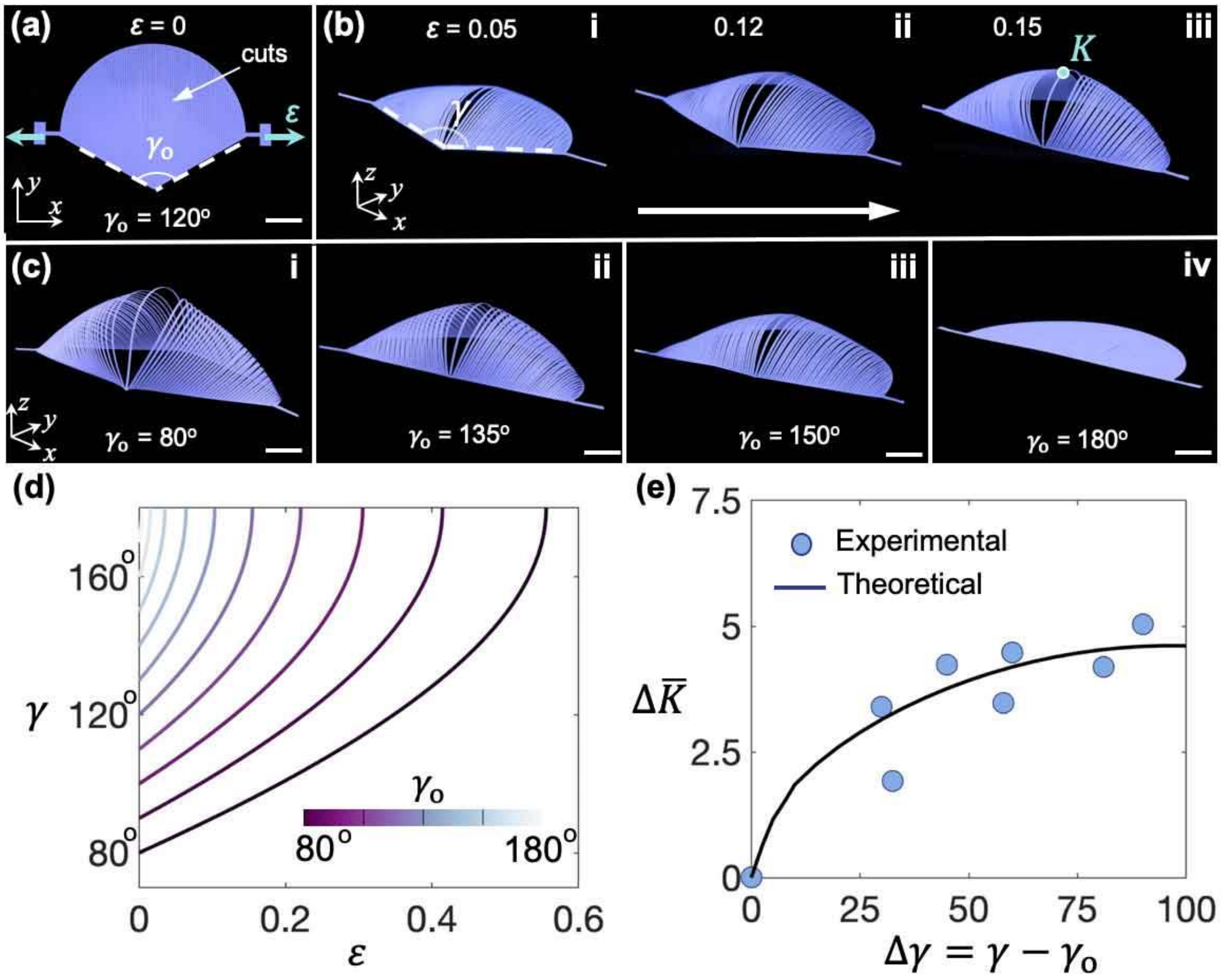

Supplementary Fig. 1 Displacement control of deployment based on an angle-γ_0 design. a Fan-shaped 2D precursor pattern with parallel cuts with the angle $\gamma_0 = 120°$ highlighted in dashed white lines. Green arrows are the direction of applied uniaxial strain ε. **b** A shell-like 3D shape formed by stretching the fan-shape 2D precursor at an applied strain ε of 0.05 (**b, i**), 0.12 (**b, ii**), and 0.15 (**b, iii**), respectively. γdenotes the varying angle in deformed states, with K being the Gaussian curvature at the top point of the shell-like shape. **c** 3D shapes formed by ultimately stretched 2D fan-shape precursors with $\gamma_0 = 80°$ (**c, i**), 135° (**c, ii**), 150° (**c, iii**), and 180° (**c, iv**), respectively. **d** Theoretical results of the varying angle g as a function of the applied strain ε in 2D precursors with different γ_0. The color bars represent 2D precursors with different γ_0. **e** Experimental and theoretical results of the variation $\Delta\overline{K}$ in the normalized Gaussian curvature of

the shell-like shape as a function of the variation $\Delta\gamma = \gamma - \gamma_o$ in the angle during deformation. Scale bars = 10 mm.

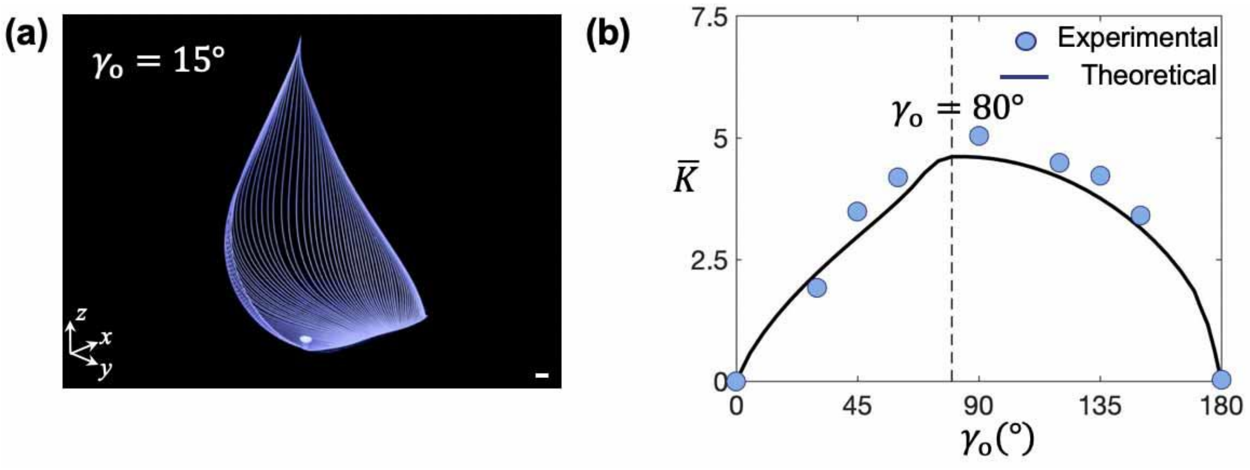

Supplementary Fig. 2 a The deployed 3D curved shapes from an ultimately stretched fan-shape 2D precursor with γ_o = 15° **b** Experimental and theoretical results of the normalized Gaussian curvature $\bar{K}$ of the ultimately stretched shell-like shape as a function of the γ_o. Scale bars = 10 mm.

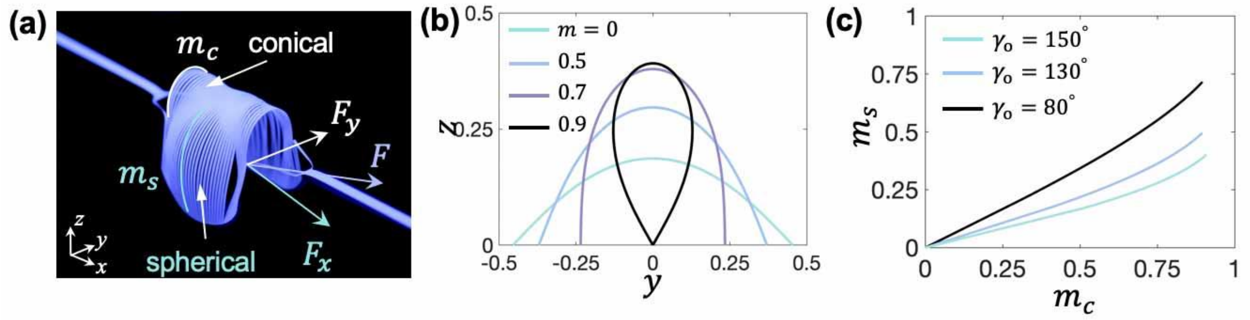

Supplementary Fig. 3 a Schematics show the force acted on the stretching ribbon. m_s and m_c denote the elliptic modulus of the ribbons in the spherical (green) and conical (white) regions, respectively. **b** Side-view morphologies of a unit-length ribbon with an increasing elliptic modulus m. **c** Theoretical result of the relationship between the elliptic modulus of two characteristic ribbons in the spherical (m_s) and conical (m_c) region of different grippers with deployment.

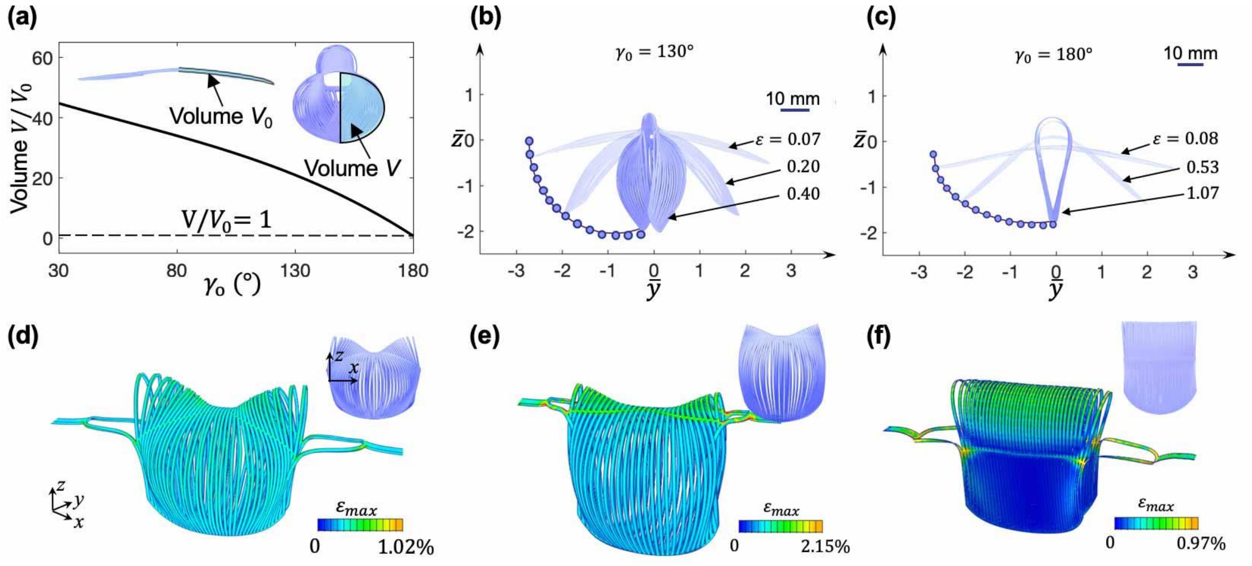

Supplementary Fig. 4 a Normalized volume V/V_0 of deployed grippers as a function of the angle γ_o in different 2D precursors. The insets show schematics of the volume in the undeployed (V_0) and deployed (V) states. Dashed black lines represent the normalized volume V/V_0 =1 in the undeformed state. **b-c** The trajectory of the gripper with γ_o = 130° and 180° with an increasing applied strain ε. **d-f** Front-view simulation results of grippers (g$_o$ = 80°, 130°, and 180°) with insets showing the experimental results. The color bar shows the maximum tensile strain in the ribbons. Scale bars = 10 mm.

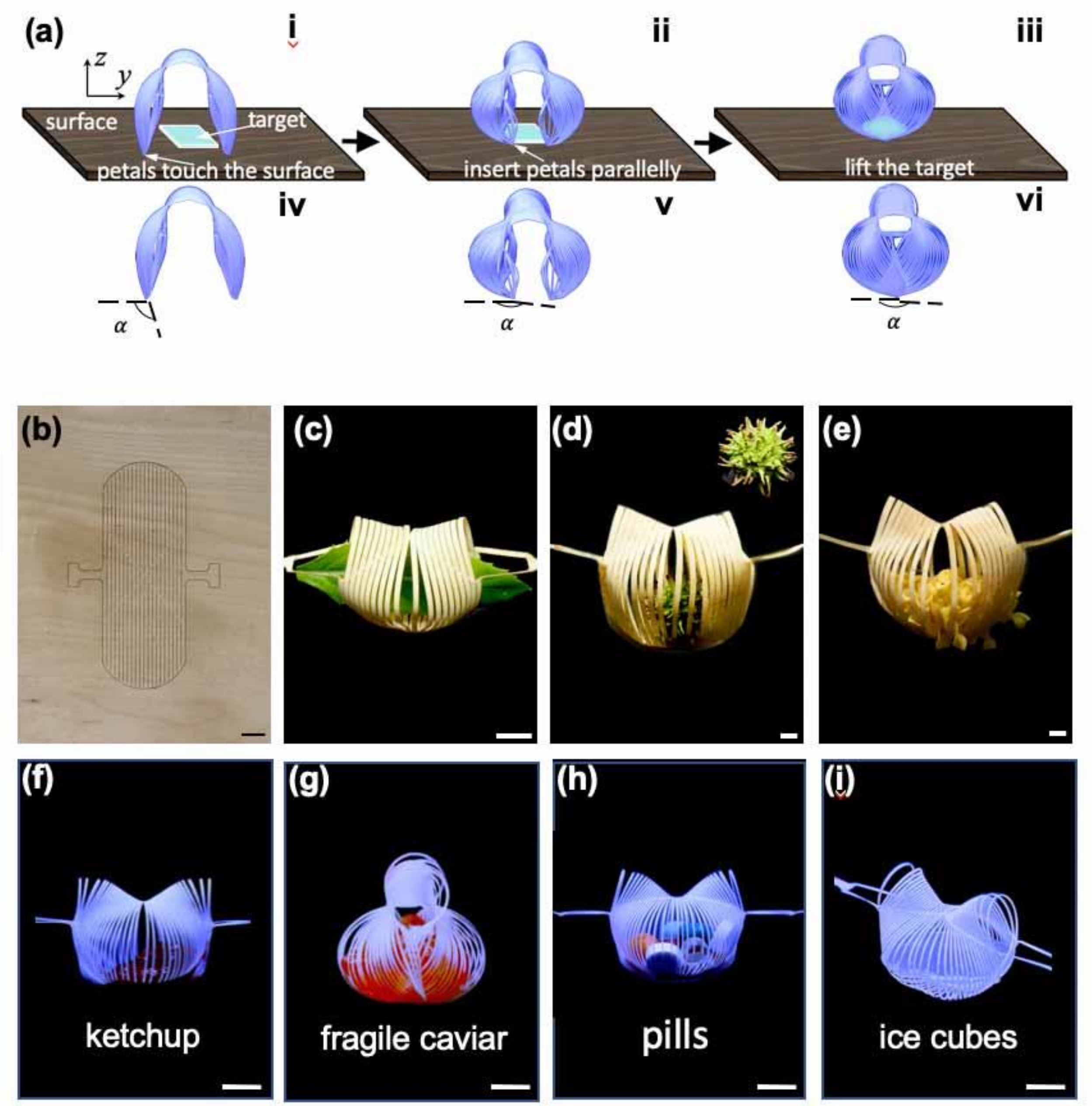

Supplementary Fig. 5 a Schematics of the gripper with γ_0 = 80° grasping an ultra-thin sheet. (**a, i**) Petals touch the lying surface. (**a, ii**) Petals affected by the compression from the surface. Petals approach the target parallelly and insert the end tip into the gap between the target and the surface. (**a, iii**) Petals lift the target. (**a, iv-vi**) Schematics show the variation in the grasping angle α in **a, i-iii**. The angle α is defined as the angle between the tangential direction at the end tip of the petal and the horizontal axis. **b-e** 2D precursor of the gripper made of a wood plate grasping a leaf (**c**), a thorny plant (**d**), and rotini (**e**). **f-i** Kirigami gripper grasping ketchup (**f**), caviar (**g**), granular pills (**h**), and ice cubes (**i**). Scale bars = 10 mm.

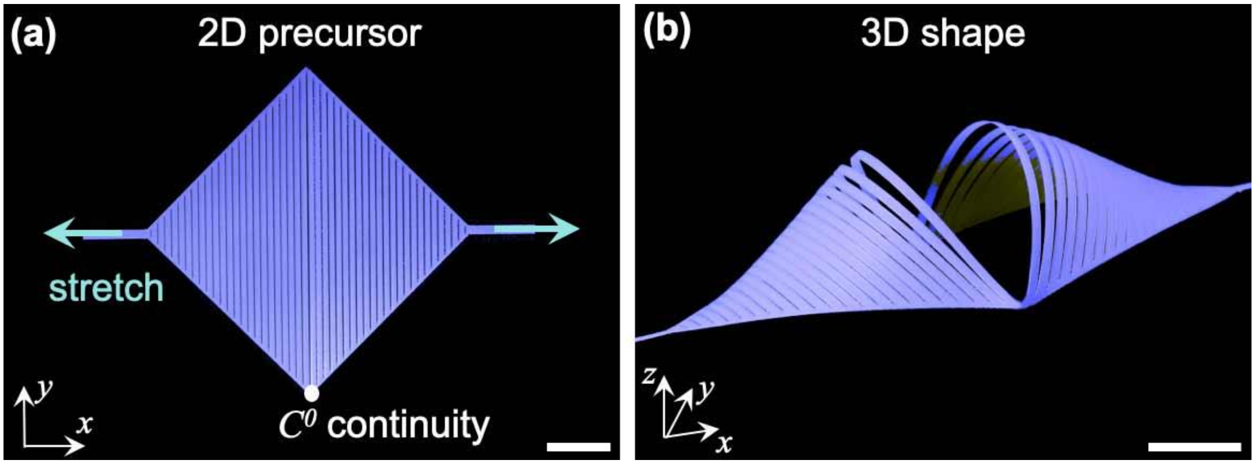

Supplementary Fig. 6 a 2D precursor with a rhombic boundary (C^0 continuity). **b** 3D shape with a gap formed by stretching the rhombic precursor. Scale bars = 10 mm.

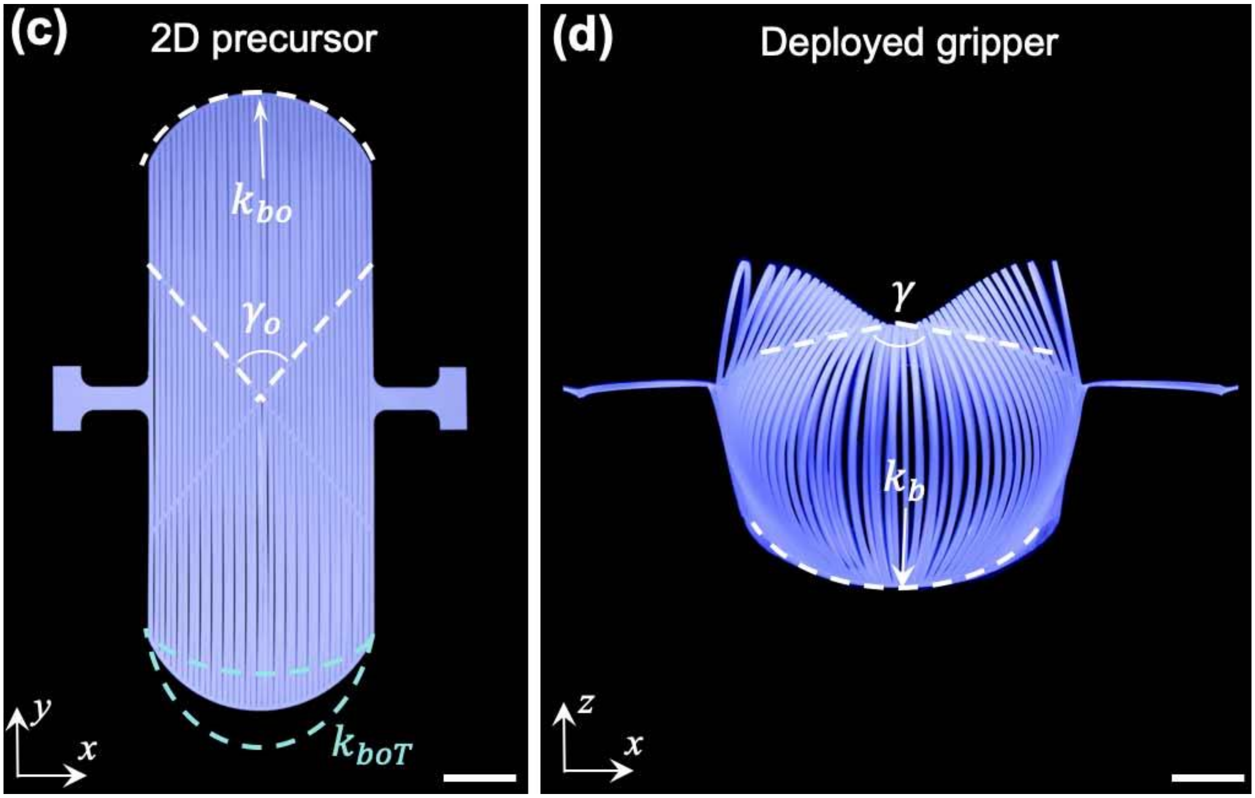

Supplementary Fig. 7 Schematics showing the variation in the curvature in the boundary and the angle. **a** 2D precursor of the gripper. γ_o is the angle in the precursor. k_{bo} denotes the prescribed curvature. k_{boT} denotes the tuned curvature in the precursor. **b** Front view of the deployed gripper. γ and k_b are the varying angle and curvature during deploying, respectively. Scale bars = 10 mm.

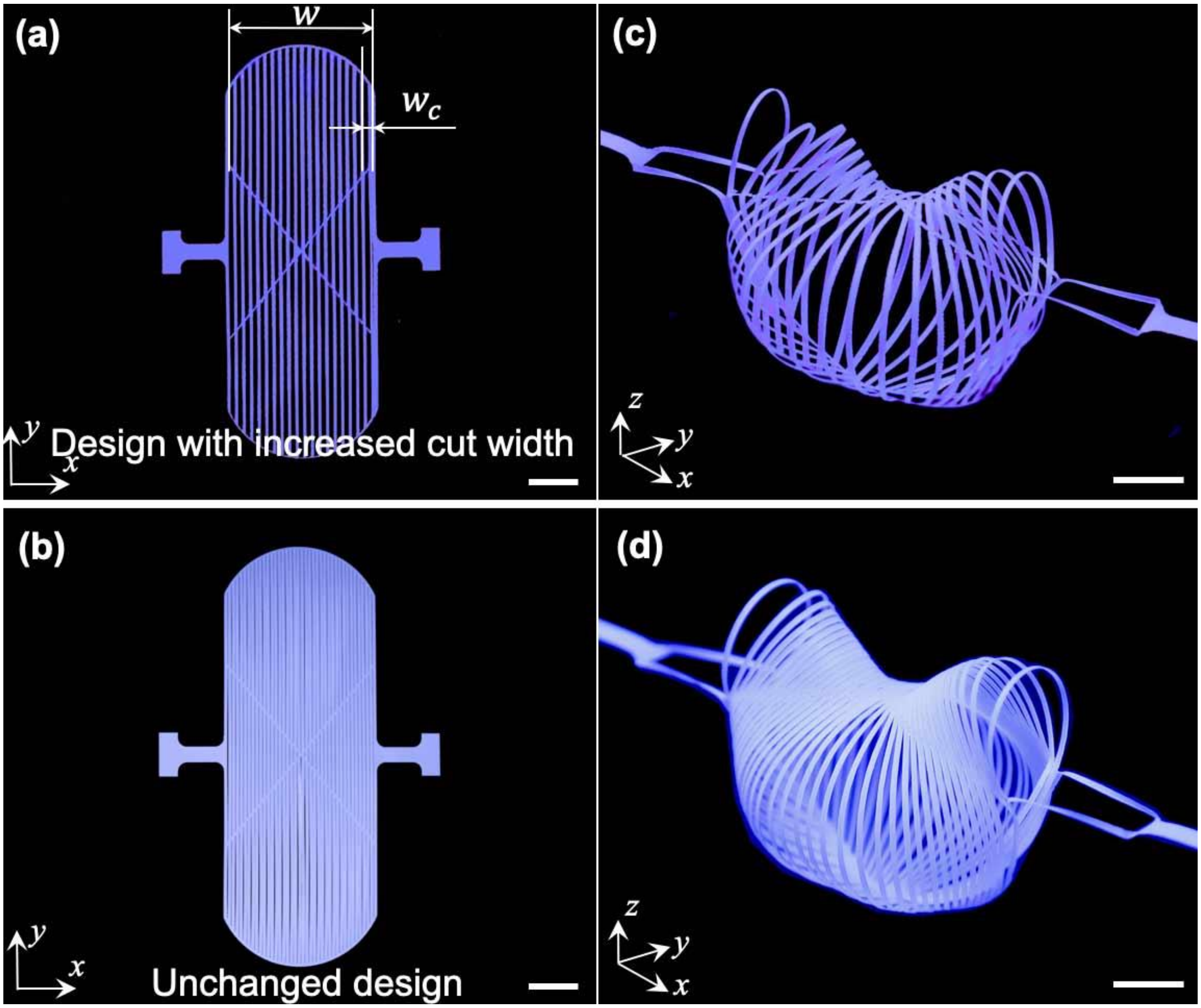

Supplementary Fig. 8 a-b 2D precursor with different cut width w_c. The w_c/w are 0.025 (**a**) and 0.003 (**b**), respectively. w_c and w denote the width of the cut and width of the gripper, respectively. **c-d** Isometric view of the deployed grippers formed by different precursors. Scale bars = 10 mm.

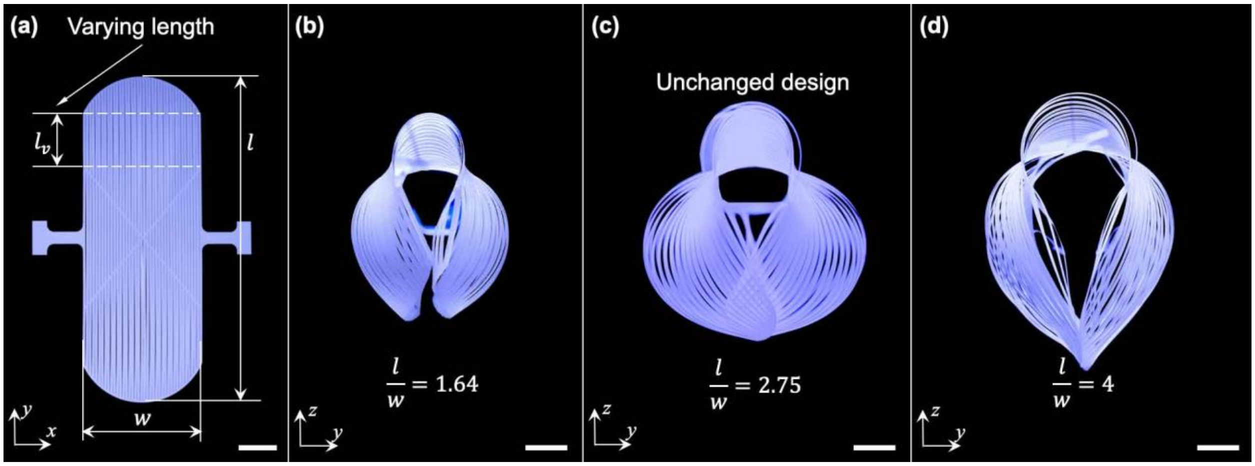

Supplementary Fig. 9 a Schematic showing the dimension of 2D precursor. l and w denote the length and the width of the gripper, respectively. l_v is the varying length to change the aspect ratio, $r_a = l/w$. **b-d** Side view of the deployed grippers with different aspect ratios. The ratios are 1.64, 2.75, and 4, respectively. Scale bars = 10 mm.

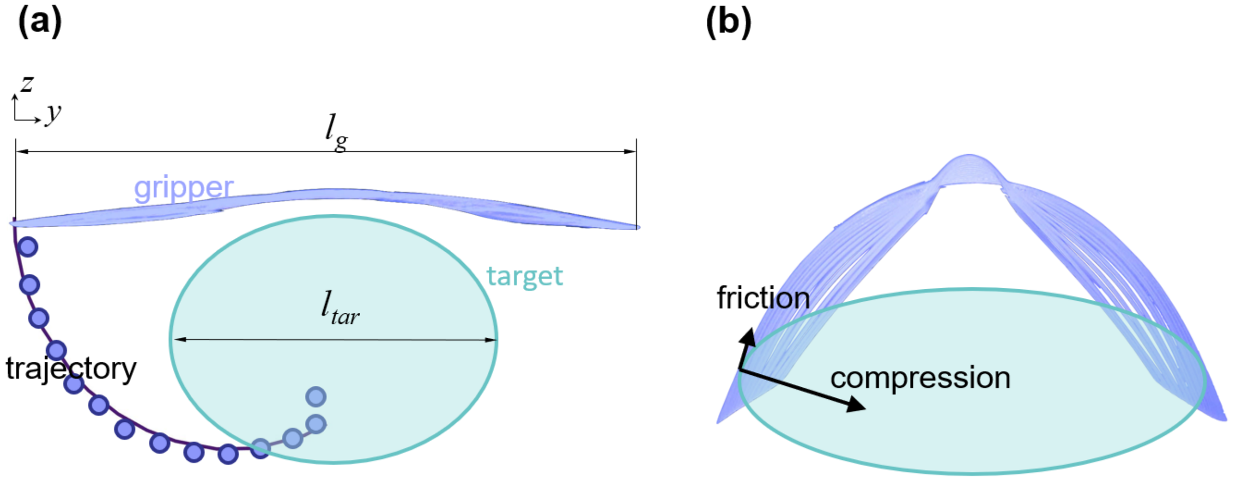

Supplementary Fig. 10 a Schematic showing the side view of the gripper encapsulating the target. l_{tar} and l_g denote the length of target and length of the undeployed gripper, respectively. The black curve and blue dots are the analytical and experimental results of the deploying trajectory, respectively. **b** Schematic showing the side view of the gripper pinching a large target. The black arrows are the compression and the friction exerted on the target by the gripper.

Supplementary Reference

1. Yang, Y., Vella, K. & Holmes, D. Grasping with kirigami shells. *Sci. Robot.* **6**, eabd6426 (2021).
2. Teoh, Z. E. *et al.* Rotary-actuated folding polyhedrons for midwater investigation of delicate marine organisms. *Sci. Robot.* **3**, eaat5276 (2018).
3. Sinatra, N. *et al.* Ultragentle manipulation of delicate structures using a soft robotic gripper. *Sci. Robot.* **4**, eaax5425 (2019).
4. Gruber, D., Wood, R. J. Advances and future outlooks in soft robotics for minimally invasive marine biology, *Science Robotics*, **7**, abm6807 (2022)
5. Roh, Y. *et al.* Vital signal sensing and manipulation of a microscale organ with a multifunctional soft gripper. *Sci. Robot.* **6**, eabi6774 (2021).
6. Song, S., Drotlef, D.-M., Son, D., Koivikko, A. & Sitti, M. Adaptive self-sealing suction-based soft robotic gripper. *Adv. Sci.* **8**, 2100641 (2021).
7. Linghu, C. *et al.* Universal SMP gripper with massive and selective capabilities for multiscaled, arbitrarily shaped objects. *Sci. Adv.* **6**, eaay5120 (2020).
8. Brown, E. *et al.* Universal robotic gripper based on the jamming of granular material. *Proc. Natl Acad. Sci.* **107**, 18809-18814 (2010).
9. Hong, Y. *et al.* Boundary curvature guided programmable shape-morphing kirigami sheets. *Nat. Commun.* **13**, 530 (2022).
10. Spivak, M. A comprehensive introduction to differential geometry, *Am. Math. Mon.* **80**, 448 (1973).
11. Love, A. E. H. A Treatise on the mathematical theory of elasticity. 4th edition, Dover Publications (2011).
