# Supplementary material for: Angle-programmed tendril-like trajectories enable a multifunctional gripper with ultradelicacy, ultrastrength, and ultraprecision
Source: Nat Commun. 2023 Aug 2;14:4625. doi: 10.1038/s41467-023-39741-6 (PMC10397260; doi:10.1038/s41467-023-39741-6)
Supplement: Supplementary file 3 — Description of Additional Supplementary Files [file 41467_2023_39741_MOESM3_ESM.pdf]

Description of Additional Supplementary Files

Supplementary Movie 1: Kirigami gripper integrated with a commercially available robotic arm manipulating various objects.

Supplementary Movie 2: Biodegradable kirigami gripper made of a leaf grasping different objects.

Supplementary Movie 3: Kirigami gripper integrated with an electromyographic prosthesis performing delicate tasks.

Supplementary Movie 4: Kirigami gripper grasping various objects actuated manually.
